# On the Employment of Nitrate of Silver as a Vesicant

**Published:** 1849-09

**Authors:** 


*On the Employment of Nitrate of Silver as a Vesicant.* By M. Delvaux.—The general action of nitrate of silver on the tissues, seems to be to separate the hydrogen. When this salt is brought in contact with an organic body, it becomes decomposed into nitric acid, oxygen, and metallic silver, in a molecular state. Silver is deposited, and this imparts to the tissue its coloration, whilst the oxygen of the oxide of silver and of the decomposed nitric acid takes up the hydrogen to form water.

When nitrate of silver is brought in contact with the skin, the effect produced varies according to the greater or smaller quantity of salt employed. If the quantity be small, it merely acts on the epidermic cells, which it disorganizes. Metallic silver is reduced to a molecular state, and combines with their elements; the epidermic tissue assumes a blackish brown coloration, owing to the metallic silver itself, and after a time the tissue is detached and drops. Where the action of the nitrate of silver is continued for a longer period of time, the true skin itself becomes affected, the effect produced varying according to whether the disorganization is merely on the surface, or more deeply seated. In the former case, an abundant serosity raises the altered epidermic surface, and produces vesication. In the latter, the true skin, being disorganized in thickness, produces an eschar.

If now, we consider that the skin varies in thickness and sensibility in different parts of the body, and according to age, sex, &c., it will be evident that a certain tact is required to regulate the quantity of nitrate of silver necessary to disorganize the epidermic layers, and procure a vesicatory effect without disorganizing the true skin. The principles by which the employment of escharotics in general is guided will suffice to prevent the occurrence of any unexpected results.

Without proceeding to enumerate all the diseases in which vesication by means of nitrate of silver may produce beneficial therapeutic effects, we will adduce a few cases in refutation of the objections that might be advanced against this form of application.

1. M. Claes, a patient in the hospital of des Viellards, who was recovering from an attack of adynamic pleuro-pneumonia with parotitis, complained, on the 3d of September, 1848, of severe pain in the left sub-scapular region, and in the lateral portion of the neck, along the trapezius muscle. The pains increased on the least movement. Exposure to a current of air had given origin to this rheumatic affection. The skin was cauterized in the sub-scapular region with a stick of nitrate of silver, moistened with water at the moment of its application. A bulla appeared in the course of an hour and a half; epidermis being removed, a slight degree of suppuration was established, and the pain entirely ceased, as if by magic, at the end of about ten hours.

The cauterized spot had been dressed immediately after cauterization with cold cream, and this was continued until the occurrence of cicatrization, which took place within the fourth day.

2. A man named Boufort, came to consult M. Uytterhoeven, at the same hospital. The old man had suffered since the preceding evening from acute stitch in the side. The pains extended along the seventh rib towards the back. As auscultation did not reveal anything abnormal in the thoracic organs, the spot to which the patient referred the pain was cauterized with nitrate of silver, previously moistened with water. The pain disappeared as the operation advanced. A vesicle was produced in the course of an hour. A compress with cerate was applied to the wound. On the following day the pleurodynia was perfectly cured without the treatment being further continued.

3. The same method was immediately employed in the case of Marie Demaitre, who had been attacked by pleurodynia in the left side of the thorax. The pain was so violent as to call forth loud cries from the patient. The cure was equally prompt and unexpected, and in the course of the day the pain entirely disappeared. The vesication was treated in the manner usually adopted in the case of ordinary vesicants.

It only remains to add a word or two on the mode of operation of this vesicant. In order to avoid all chance of irregularity, it is necessary to rub the whole surface on which vesication is to be induced, lightly but equally with the point of the stick moistened with a drop of water, and to continue long enough until a gray coloration is produced. This effect is generally obtained in a minute and a half. If a deeper action be required, owing to the thickness of the epidermis, or a more strongly marked therapeutic effect be sought, the operation must be repeated over the same surface and with the same precautions.

M. V. Uytterhoeven has always found this vesicant answer his expectations most fully, both in private practice and in the wards of the hospital des Viellards.—*Monthly Retrospect*, June, 1849, from *Nouvelliste Médicale Belge.*

